# Linking health worker motivation with their stated job preferences: A hybrid choice analysis in Ethiopia

**DOI:** 10.1016/j.socscimed.2022.115151

**Published:** 2022-06-20

**Authors:** Nikita Arora, Romain Crastes dit Sourd, Kara Hanson, Dorka Woldesenbet, Abiy Seifu, Matthew Quaife

**Affiliations:** a Faculty of Public Health and Policy, London School of Hygiene and Tropical Medicine, UK; b Centre for Decision Research, Management Division, Leeds University Business School, UK; c School of Public Health, Addis Ababa University, Ethiopia

**Keywords:** Discrete Choice Experiments, Stated preferences, Hybrid choice analysis, Health workers, Motivation

## 1 Introduction

Skilled, knowledgeable, and productive health workers are important for a well-functioning health system (World Health Organization, 2010), equitable access to which is crucial to meet the Sustainable Development Goals (Lassi et al., 2016). Understanding the job preferences of health workers can help policymakers better align incentives and retain a motivated workforce in the public sector, improving the quality and sustainability of healthcare delivery (Lagarde, 2013; Lagarde & Blaauw, 2009; Lindelow & Serneels, 2006).

Discrete choice experiments (DCEs) are a popular method in health economics used to determine the driving factors behind the relative preferences of health workers for different job attributes, that either can’t be observed in real life or service characteristics that haven’t yet been introduced. The aim of such DCEs is that findings can be leveraged by policy makers to improve health worker retention and productivity in exchange for the right incentives (Lagarde & Cairns, 2012; Mandeville et al., 2016; Mangham & Hanson, 2008; Saran et al., 2020). For example, one study evaluated the relative importance of material and non-material policy incentives in the motivation and retention of community health workers in Western Kenya, using a DCE (Saran et al., 2020). The study showed that community health workers did not just care about salary, but non-material job aspects like appreciation from the community and health facility staff. The DCE, however, did not report whether the health workers most preferring salary had different motivations in comparison to those valuing community appreciation more.

When designing DCEs that can be policy relevant and appeal to the decisions of health workers, a key consideration should also be to incorporate elements of the respondents’ cognitive process which have been identified as important in decision making (McFadden, 1986). Based on a behavioural approach to choice, McFadden illustrated that the process of making a choice, and choice itself, can be better understood if models can combine *‘hard information’* such as well measured socio-economic characteristics of respondents’ with *‘soft information’* such as indicators of their psychological processes such as attitudes, motivation and affect (McFadden, 2001). The motivation intensity approach defines motivation as a “set of energetic forces that originate both within as well as beyond an individual’s being, to initiate work-related behaviour and to determine its form, direction, intensity, and duration” (Pinder, 2014). Further, self-determination theory (Deci & Ryan, 1985; Lohmann et al., 2017) distinguishes between two key dimensions of motivation - extrinsic and intrinsic – both of which are important determinants of what invigorates people to work and refer to motivation driven by external recognition and internal enjoyment for doing the activity, respectively. While improving health worker motivation is known to be a key mechanism for achieving health impact, by encouraging health providers to exert more effort in return for the right incentives (Borghi et al., 2018b; Quaife et al., 2021), in DCEs motivation has never been incorporated as an antecedent to study health worker’s job choices.

This paper argues that the overall motivation of health workers, alongside the extent to which they may be extrinsically or intrinsically motivated, influences the job attributes they value, and therefore the utility a given job would provide to them. Motivation is therefore considered a source of variation in preferences among health workers. Including motivation directly into the specification of the utility function of the choice model, such as in the form of an interaction term, is theoretically flawed because of the risk of endogeneity bias and measurement error (Bahamonde-Birke & Ortúzar, 2014; Ben-Akiva & Bierlaire, 1999; Bolduc & Alvarez-Daziano, 2010; Kløjgaard & Hess, 2014; McFadden, 1986; Raveau et al., 2010). Studies that include attitudes and perceptions in the analysis of choice thus tend to use hybrid choice models which allow psychological constructs to be included as latent variables (Kløjgaard & Hess, 2014; Santos et al., 2011).

In this study, we demonstrate the application of a hybrid choice model in understanding the job preferences of community health workers in Ethiopia, also known as health extension workers (HEWs), using motivation as a latent variable. HEWs are responsible for the delivery of 16 primary healthcare interventions, predominantly in rural areas, ranging from preventive services in family planning and immunizations to basic curative services for communicable and some non-communicable diseases (Arora et al., 2020). They account for the second largest health workforce in Ethiopia with close to 21% of the recurrent government health expenditure invested in their salaries (Wang et al., 2016). We believe that a better understanding of how HEWs make trade-offs between attributes of their jobs can inform policy decisions aimed at overcoming the gradual rise in the rate of attrition within this cadre (MERQ Consultancy Plc, 2019).

This study fills two key gaps in the literature on health worker behaviour and preferences. First, to our knowledge, no DCE to date has looked into how motivation of health workers could be a source of preference heterogeneity within stated preferences methods, the knowledge of which can be leveraged by managers to get these health workers to exert more effort in return for the right incentives. Further, previous studies analysing health workers’ preferences have been limited by focussing only on highly skilled health workers, such as doctors and nurses, while not including lower skilled health workers such as community health workers who deliver the majority of primary health care services in countries like Ethiopia.

## 2 Data and Dce Design

### 2.1 Data

We used data from a survey designed to quantitatively assess the job preferences of different cadres of health workers based in four regions of Ethiopia: Tigray, Amhara, Oromia and Southern Nations Nationalities and People (SNNP). The DCE was embedded within an endline survey collecting information for the process evaluation of a quality improvement programme implemented by the Ministry of Health in Ethiopia, conducted in June 2019. More details about the study can be found in Quaife et al (2021) and Lamba et al (2021). The sample consisted of a cadre-stratified random sample of 404 middle and lower-skilled health workers in the Ethiopian health system, including data on three cadres – HEWs, non patient-facing staff like health facility administrators, and mid-level maternal and new-born healthcare providers including nurses and midwives. In this paper, since our aim was to focus on understanding the preferences of lower-skilled health workers, and since Lamba et al. (2021) had reported heterogeneity in preferences between the three health worker cadres being studied, we only used data on HEWs (n=202) who comprised half the survey sample. In addition to the DCE, the survey also collected information on various respondent sociodemographic characteristics. Table 1 provides descriptive statistics for selected sociodemographic characteristics.

#### Motivation instrument

The survey also collected information about the respondent’s motivation to do their jobs. To measure motivation, the survey adapted a quantitative tool which was developed and validated among community health workers in Uganda (Eichler & Levine, 2009), with small changes to wording made to suit the Ethiopian context. This tool consisted of 17 questions, with eight further questions added from a health worker motivation evaluation conducted in Tanzania (Borghi et al., 2018a) to explore extrinsically motivating factors in further depth. Finally, with input from senior staff implementing the QI programme five more questions were added around activities which were part of the programme, relating to training and recognition for doing a good job, taking the total number of questions to 30. All statements had Likert scale response options where 1=strongly agree, 2=agree, 3=neutral, 4=disagree, 5=strongly disagree.

A team of seven, trained research assistants from School of Public Health, Addis Ababa University administered the survey face-to-face with the respondents, in Amharic, Tigrigna, and Oromifa languages using Open Data Kit (https://opendatakit.org) software on tablet computers. Informed consent was obtained from all participants before data were collected, and the study was undertaken with ethical approval from the Observational Research Ethics Committee of the London School of Hygiene and Tropical Medicine and a program evaluation waiver from the Ethics Committee of the Ethiopian Public Health Association.

### 2.2 DCE development and design

The DCE had six job attributes, identified after a thorough review of literature on health workforce discrete choice experiments conducted in the East African context (Blaauw et al., 2013; Mandeville et al., 2017; Mangham & Hanson, 2008; Rockers et al., 2012). Further details on the process of selection of attributes are reported in Lamba et al (2021); Table 2 provides the list of attributes and levels. The DCE was piloted among 19 district health office staff in December 2017, before the baseline survey for the main study was conducted. The pilot had a ten-task fractional factorial design while the final was a seven-task, D-optimal design based on priors from the pilot, conducted in NGENE (Choice Metrics, 2012). The design was main effects only, and because it was a subsection of a survey which took a relatively long time to complete, design diagnostics indicated that we were able to reduce respondent burden through reducing the number of tasks from 10 to seven. In each task the participants were presented with two job alternatives representing a generic health worker’s job.

**Figure 1:**
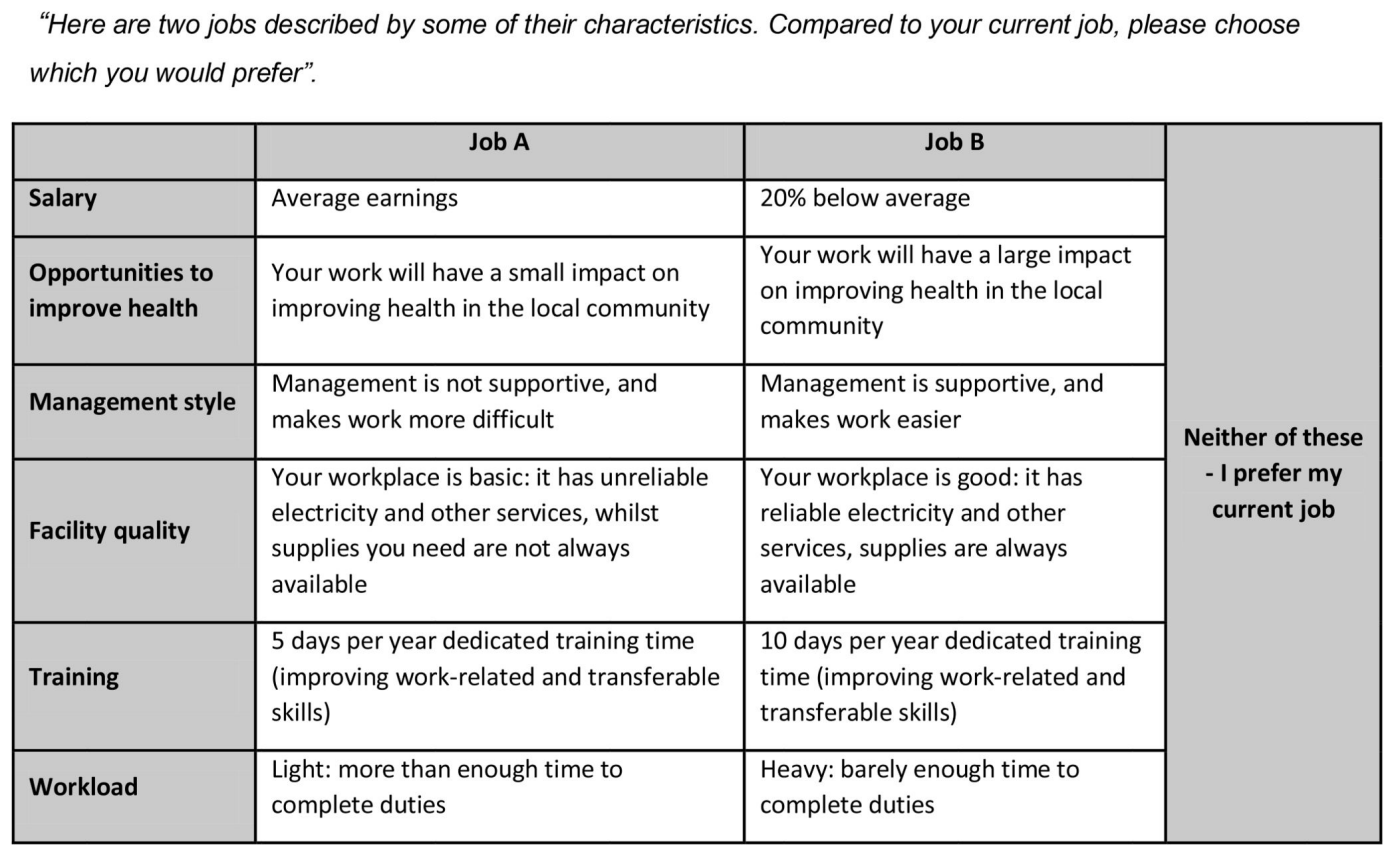
Example choice task

To increase realism and allow for the estimation of unconditional demand, a generic opt-out alternative was included, modelled simply as a constant with no attribute levels, representing the choice of picking neither of the presented job profiles and staying in their current job. Figure 1 shows an example of how choice tasks were presented to respondents. We used Apollo version 0.2.5 (Hess & Palma, 2019) in R (version 4.0.2) to analyse our data.

## 3 Methods

### 3.1 Modelling framework

Standard random utility models estimated on DCE data are based on the notion that respondent choice is determined by the utilities that they perceive for the given alternatives. For respondent *n*, alternative *i*, and choice situation *t*, this utility, *U*, can be given by

[1] $$U_{i,n,t}=V_{i,n,t}+\epsilon_{i,n,t}$$

It is made up of a modelled component *V_i,n,t_* and a random component *ϵ_i,n,t_* which follows a type 1 extreme value distribution. Further, we have:

[2] $$V_{i,n,t}=\beta_{n}^{\prime }x_{i,n,t}$$

Where *β_n_* is a vector of taste coefficients and *x_i,n,t_* is a vector of attributes for alternative *i*, which can include alternative specific constants (ASCs) for all but one of the alternatives. Given the assumptions about the error term, the probability that respondent *n* chooses a given alternative *i* conditional on *β_n_* and the ASCs in choice situation *t* corresponds to the well-known multinomial logit model structure:

[3] $$P_{i,n,t}=\frac{e^{V_{i,n,t}}}{\sum_{j=1}^{J}V_{j,n,t}}$$

The elements in *β_n_* can be allowed to vary across respondents, either based on their observed characteristics (by adding “interaction variables”) or randomly by using a joint distribution *f*(*β_n_* | Ω) where Ω is a vector of parameters to be estimated, relating to the means and covariance structures of the elements in *β_n_*. This leads the model to capture random heterogeneity in preferences.

A share of the variance of random taste heterogeneity for job characteristics can be linked to random variations in *motivation* by the means of a hybrid choice model structure. Data on psychological constructs such as motivation come from answers to psychometric tools comprising of attitudinal statements which cannot be treated as direct measures of the attitude itself and are prone to measurement error. As such, these constructs can’t be included directly in the utility function of choice models as interaction variables and need to be treated differently.

**Figure 2:**
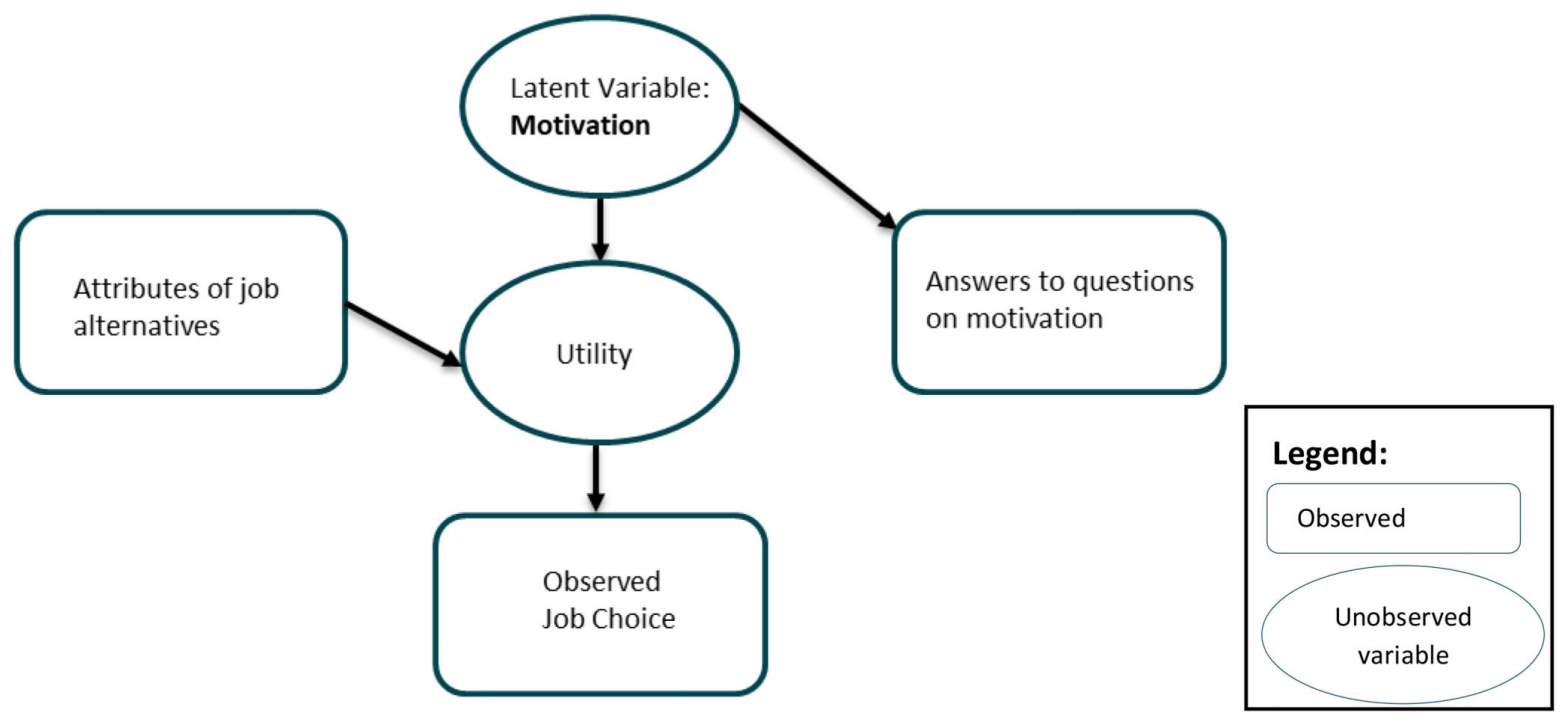
Study hybrid choice model structure

The hybrid choice framework provides a way to accommodate such psychological constructs by jointly modelling the responses to the stated choice component as well as to attitudinal questions, as illustrated by Figure 2.

In other words, this modelling framework suggests that answers to attitudinal questions should be treated as dependent rather than explanatory variables (Ben-Akiva et al., 2002), and in addition to the attributes of job alternatives, motivation can be used as a latent variable (or a series of latent variables related to different aspects of motivation) to explain the relationship between the observed job choices of community health workers and the answers to a series of questions related to respondent motivation.

In the context of this paper, this modelling framework allows us to disentangle the share of unobserved heterogeneity in respondent preferences for job attributes which is related to random variations in taste, from the share which is related to random variations in motivation across respondents. This gives richer behavioural insights. We conducted exploratory factor analysis to identify the correlation between statements included in the motivation tool and to assess the number of latent variables that can be included in our hybrid choice model. This is explained in further detail in the following section.

### 3.2 Factor analysis of motivation measure

For our factor analysis, we used the covariance between variables to identify distinct underlying groups of variables which are correlated with one another. This made it possible for us to understand the dimensionality of the motivation measure and the main statements explaining each dimension. Each individual factor was incorporated as a separate latent variable in the hybrid choice model.

Overall, three factors were revealed to be statistically significant and for each of them, the representative statements were also identified. Only 24 out of 30 statements passed our criteria of inclusion^i^ and the rest were dropped. The factors along with their statements and factor loadings are given in Table 6 in the appendix. We assigned qualitative titles to the identified factors based on the statements that characterised each one. These three factors corresponded to the latent variables (*α*_1–3_) included in the model and are described below:

*α*_1_ : Intrinsic motivation (driven by reaching personal and professional goals)

*α*_2_ : General contentment with the job

*α*_3_ : Extrinsic motivation (driven by external recognition)

### 3.3 Model specifications

We estimated three main models to demonstrate a gradual build-up of model complexity. We started with a main effects Multinomial Logit (MNL) model, followed by allowing for random heterogeneity in preferences by estimating a main effects Mixed Multinomial Logit (MMNL) model. Finally, to measure the relationships between motivation and respondent preferences, we estimated a Hybrid Choice Model (HCM), where motivation enters our model as a series of latent variables.

To get around the issues with local optima, we ran all models with different sets of starting values, obtained through the analysis of appropriate base models (Kløjgaard & Hess, 2014). As we have more than five attributes in our DCE, we used 2,000 MLHS draws (Czajkowski & Budziński, 2019).

#### 3.3.1 MNL and MMNL

We used the specification below to parameterise the MNL:

[4] $$V_{n,j,t}=\beta_{\text{asc}\;}+\beta_{\text{salary}\;\text{avg}\;}\cdot x_{\text{salary}\;\text{avg}\;,j}+\beta_{\text{salary}\;\text{plus}\;}\cdot x_{\text{salary}\;\text{plus}\;,j}+\beta_{\text{good}\;\text{mgmt}\;}.x_{\text{good}\;\text{mgmt}\;,j}+\beta_{\text{good}\;\text{facility}\;}\cdot x_{\text{good}\;\text{facility}\;,j}+\beta_{\text{training}\;5}\cdot x_{\text{training}\;5,j}+\beta_{\text{training}\;10}\cdot x_{\text{training}\;10,j}+\beta_{\text{medium}\;\text{workload}\;}\cdot x_{\text{medium}\;\text{workload}\;,j}+\beta_{\text{heavy}\;\text{workload}\;}\cdot x_{\text{heavy}\;\text{workload}\;,j}+\beta_{\text{good}\;\text{impact}\;}\cdot x_{\text{good}\;\text{impact}\;,j}$$

Where *j* = *A, B* for the two job alternatives at choice situation *t*, *β_asc_* is the alternative specific constant (ASC) for the job, and different *β_S_* represent the parameters for each attribute level used to characterise job alternatives included in the DCE. We dummy coded all attributes where the base level was fixed to zero. Our choice for which alternative should be used as a base for the ASC, as well as the base selection for other attributes followed choice modelling literature in the choice of normalization for alternative-specific constants and categorical variables by deliberately over-specifying the model (attempting to estimate all parameters) and then omitting those with the lowest variance (Walker, 2001). On the basis of this, we normalized to zero the constant for job A, less than average salary, no days of training and low workload. The opt-out was parametrised with just an ASC.

Further, to allow for random heterogeneity in respondent preferences, we estimated an MMNL, such that the utility derived from a given attribute level (taking *salary average* as an example) was now given by:

[5] $$\beta_{\text{salary}\;\text{average}\;}=\mu_{\text{salary}\;\text{average}\;}+\sigma_{\text{salary}\;\text{average}\;}\cdot \eta_{\text{salary}\;\text{average,}\;n}$$

Where *η_salary average,n_* indicates a vector of draws coming from a standard normal distribution *N* ~ (0,1). All attributes were assumed to be randomly distributed, and except *heavy workload,* they were all specified to follow a normal distribution. *Heavy workload* was set as a negative *μ*-shifted log-normal distribution (Crastes dit Sourd, 2021) to acknowledge literature on the negative effects of long term heavy workload and to recognise that the majority of respondents in our dataset were either neutral to a heavy workload or showed disutility towards it (Kc et al., 2020; Trivellas et al., 2013). It’s worth noting that the assumption of independence of random parameters comes at the cost of not being able to account for scale effects; a necessity given the computational concerns around a heavily parameterized specification.

#### 3.3.2 The Hybrid Choice Model

The specification of the HCM can broadly be broken down in three components: the specification of the structural equation of the latent variable, specification of the measurement model, and specification of the utility function in the choice model component.

##### Structural equation of the latent variable

We used three latent variables in our model, relating to the three dimensions of motivation. The structural equation for each latent variable *l* can simply be written as *α_l,n_*, which indicates a vector of draws coming from a standard normal distribution *N* ~ (0,1).

##### The measurement model

As explained above, by using an exploratory factor analysis on the 30 indicators in the motivation tool we were able to identify 24 indicators with factor loadings >0.35, followed by identifying three factors indicating different dimensions of motivation. As given in Table 6 in the appendix, thirteen indicator statements loaded to the first factor, eleven loaded to the second factor and two statements loaded to the third factor. Further, in an attempt to reduce issues during model estimation, while ensuring that all the latent variables were identified, we only used the indicators featuring the highest factor loadings for each one of the three factors. The *k* statements on motivation used in the final models are reported in Table 3. A total number of 7 statements were used in the modelling work.

We used an ordered logit specification for all 7 indicator questions (with *s* levels each), in line with the approach advocated by Daly et al. for ordinal indicators (Daly et al., 2012). The likelihood for observing a given value *s* for indicator *I* linked to latent variable *I* corresponds to :

[6] $$P_{I_{k,n}}=\sum_{s=1}^{S}\left(I_{k,n}==s\right)\left[\frac{e^{\tau_{k,s}-\zeta_{k}\alpha_{l,n}}}{1+e^{\tau_{k,s}-\zeta_{k}\alpha_{l,n}}}-\frac{e^{\tau_{k,s-1}-\zeta_{k}\alpha_{l,n}}}{1+e^{\tau_{k,s-1}-\zeta_{k}\alpha_{l,n}}}\right]$$

Where *ζ_k_* measures the impact of a given latent variable on indicator *I* and where *τ_k,s_* with *s* = 0,…,5 are a set of estimated threshold parameters where *τ*_*k*,0_ = –∞ and *τ*_*k*,5_ = +∞ for normalisation purposes. The selected indicator statements within each latent variable, along with the expected relationship between *I_k_n__* and *α_n,k_* are given in Table 3.

Since some indicator statements were positively framed while others were negatively framed, a positive value for *ζ_k_* in the above equation would mean that as *α_n,k_* increases, the likelihood of a higher value for *I_k_n__* decreases for positively framed statements and opposite for negatively framed statements (Kløjgaard & Hess, 2014).

##### Specification of utility in the choice model component of the hybrid choice model

The utility derived from a given attribute *β* (taking *salary average* as an example and omitting subscripts for clarity) now becomes the following for the HCM:

[7] $$\beta_{\text{salary}\;\text{average}\;}=\mu_{\text{salary}\;\text{average}\;}+\sigma_{\text{salary}\;\text{average}\;}\eta_{\text{salary}\;\text{average}\;}+\theta 1_{\text{salary}\;\text{average}\;}\alpha_{1}+\theta 2_{\text{salary}\;\text{average}\;}\alpha_{2}+\theta 3_{\text{salary}\;\text{average}\;}\alpha_{3}$$

The parameters labelled as *θ* capture the effect of the latent variables *α*_1_, *α*_2_ and *α*_3_ on preferences. Interpreting the results requires the reader to look jointly at the sign and magnitude of the *θ* parameters as well as the *ζ* parameters introduced in Equation 6 and Table 3. This is further detailed in the results section where we show how the variations in *α*_1_, *α*_2_ and *α*_3_ (which again, are normally distributed with a mean of 0) jointly affect the choice model and the indicator, giving rise to a hybrid model. This model now jointly maximises the likelihood of observing the choices made by each respondent (*choice model component*) and the likelihood of observing each of the seven statements on motivation (*measurement models component*). Given the many distributional assumptions made, simulation methods are used for estimating the parameters and all models were estimated using 2,000 MLHS draws. The likelihood function of the hybrid choice model corresponds to:

[8] $$LL(\Omega_{\beta },\theta ,\zeta ,\tau )=\sum_{n=1}^{N}\ln\int_{\beta }\int_{\alpha }\prod_{t=1}^{T}P_{nt}\cdot \prod_{k=1}^{K}P_{I_{k,n}}f(\beta_{n}|\Omega )g(\alpha )\delta \beta \delta \alpha$$

This is different than the likelihood for a corresponding MMNL, where the three latent variables *α*_1_, *α*_2_ and *α*_3_ do not influence taste heterogeneity and where there is no measurement model:

[9] $$LL(\Omega_{\beta })=\sum_{n=1}^{N}\ln\int_{\beta }\prod_{t=1}^{T}P_{nt}f(\beta_{n}|\Omega )\delta \beta$$

## 4 Results

Results from the three main models show that as random heterogeneity in respondent preferences is incorporated in the MMNL, model fit improves in comparison to the MNL. This was as expected because the MNL is quite restrictive and does not allow for the heterogenous preferences of respondents. Table 4 gives the model goodness of fit.

We also see that the signs of all attributes were consistent between the three models, which was reassuring in establishing the general fit of our data with the models. The results of the MNL and MMNL are given in Supplementary file 1. Further, a reduced form hybrid choice model was also estimated in order to assess if he log-likelihood at convergence for such a model was different than the log-likelihood of the choice model component of the HCM at convergence.

### 4.1 Estimation results

Table 5 shows the estimation results of the HCM. These are informative in understanding how the three dimensions of HEW motivation, given by the three latent variables, affect their preferences for different job attributes.

We start with *α*1 (representing *intrinsic motivation*) and focus on the parameters labelled as θ and ζ, which we interpret based on the direction of association between indicator questions and the latent variables given in Table 3. We find that HEWs who agreed to the three motivational statements informing *α*1, indicating intrinsic motivation, were also more likely to prefer jobs that offered less days of training, good facility quality, and good health outcomes. They show dislike towards 20% more than average salaries. Looking at parameters related to *α*2 (representing *general contentment with job*), we find that the respondents who are more likely to agree with *“I am proud of the work I do”* and *“In general I am satisfied with my role”* have lower preferences for less days of training and care less about heavy workloads. Finally, results about *α*3 (representing *extrinsic motivation)* show that HEWs who are more likely to agree with “*at the moment I don’t feel like working as hard as I can”* have stronger disutility for a heavy workload and preferences for good facility quality. At the same time, respondents who are more likely to agree with *“I am strongly motivated by the recognition I get from other people”* have opposite preferences, that is they experience less disutility from heavy workload and less utility from good facility quality.

We further demonstrate the association between multidimensional motivation and HEW preferences, by plotting the correlation between respondent preferences for 20% more than average salary and the parameters which capture the effect of the latent variables *α*_1_ (intrinsic motivation), *α*_3_ (extrinsic motivation) on their preferences for the attribute level. We also plot the correlation between preferences for a heavy workload and the *θ* parameters for the two latent variables. In line with the literature, we hypothesise that extrinsically motivated people will prefer a higher than average salary and dislike a heavier workload, while people with intrinsic motivation will not care too much about a heavy workload and not prefer a higher salary (Deci & Ryan, 1985). Most of the results for latent variable 2, depicting general contentment with HEW jobs were found to be statistically insignificant.

**Figure 3:**
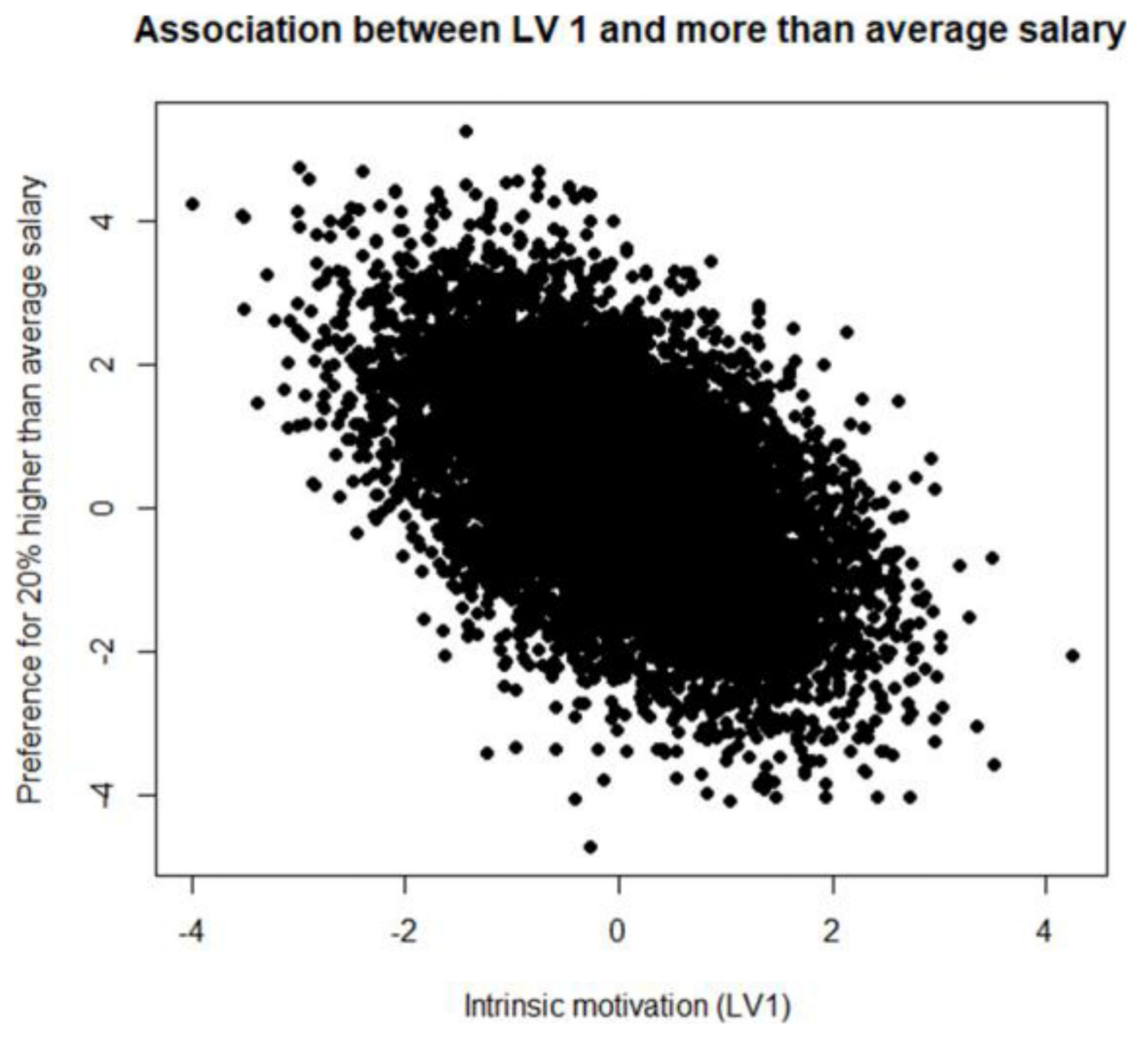
Association between intrinsic Motivation and a higher than average salary

**Figure 4:**
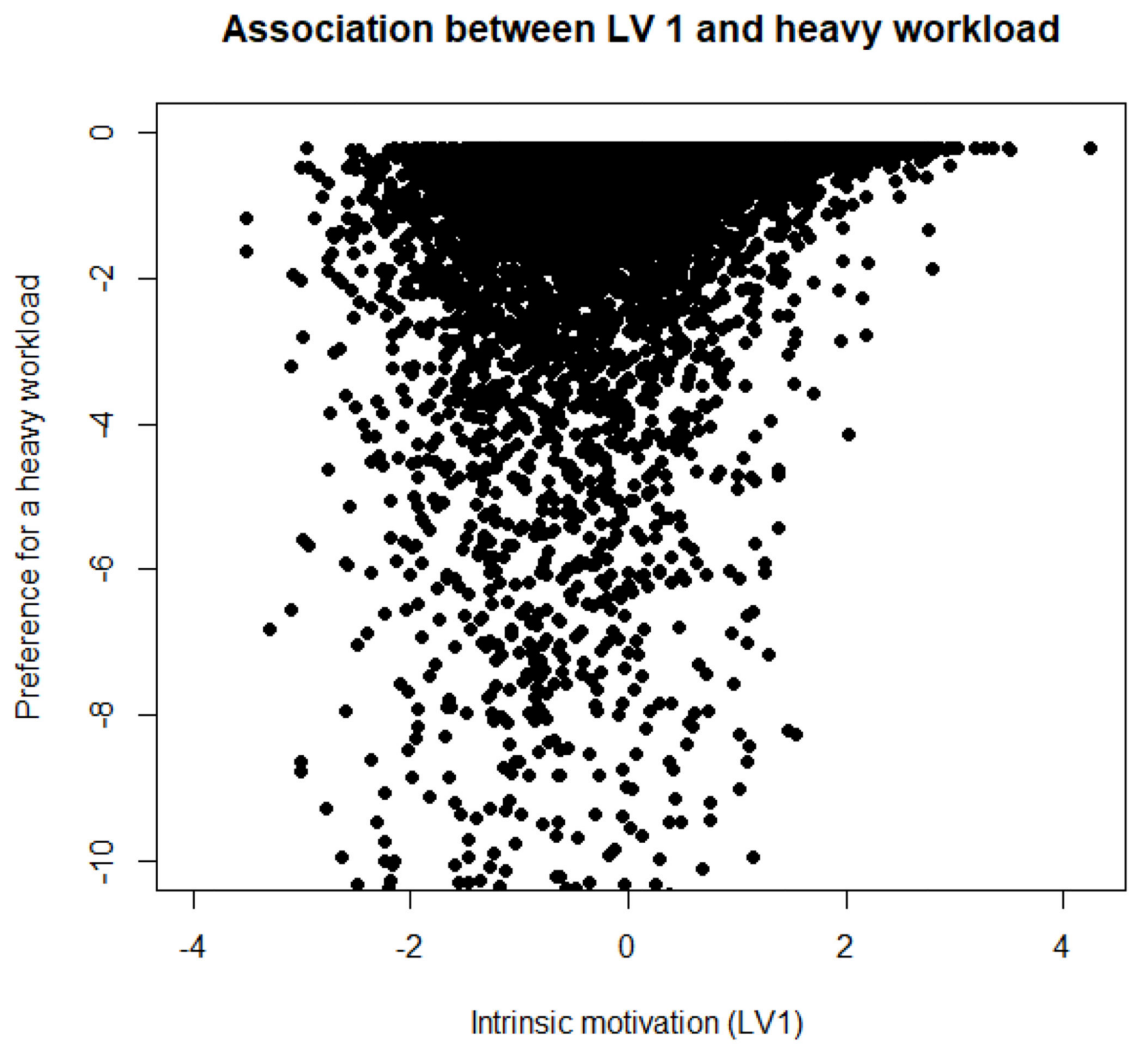
Association between extrinsic motivation and a heavy workload

Figure 3 shows the direction in which intrinsic motivation affects HEW’s preferences for a higher than average salary. We see that as HEWs become more intrinsically motivated, their preferences for a higher average salary decrease. Figure 4 shows the same association but for a heavy workload. We see that as intrinsic motivation diminishes (goes from 0 to -4), preferences for a heavy workload also reduce, however, with a rise in intrinsic motivation, HEWs become more neutral to a heavy workload.

**Figure 5:**
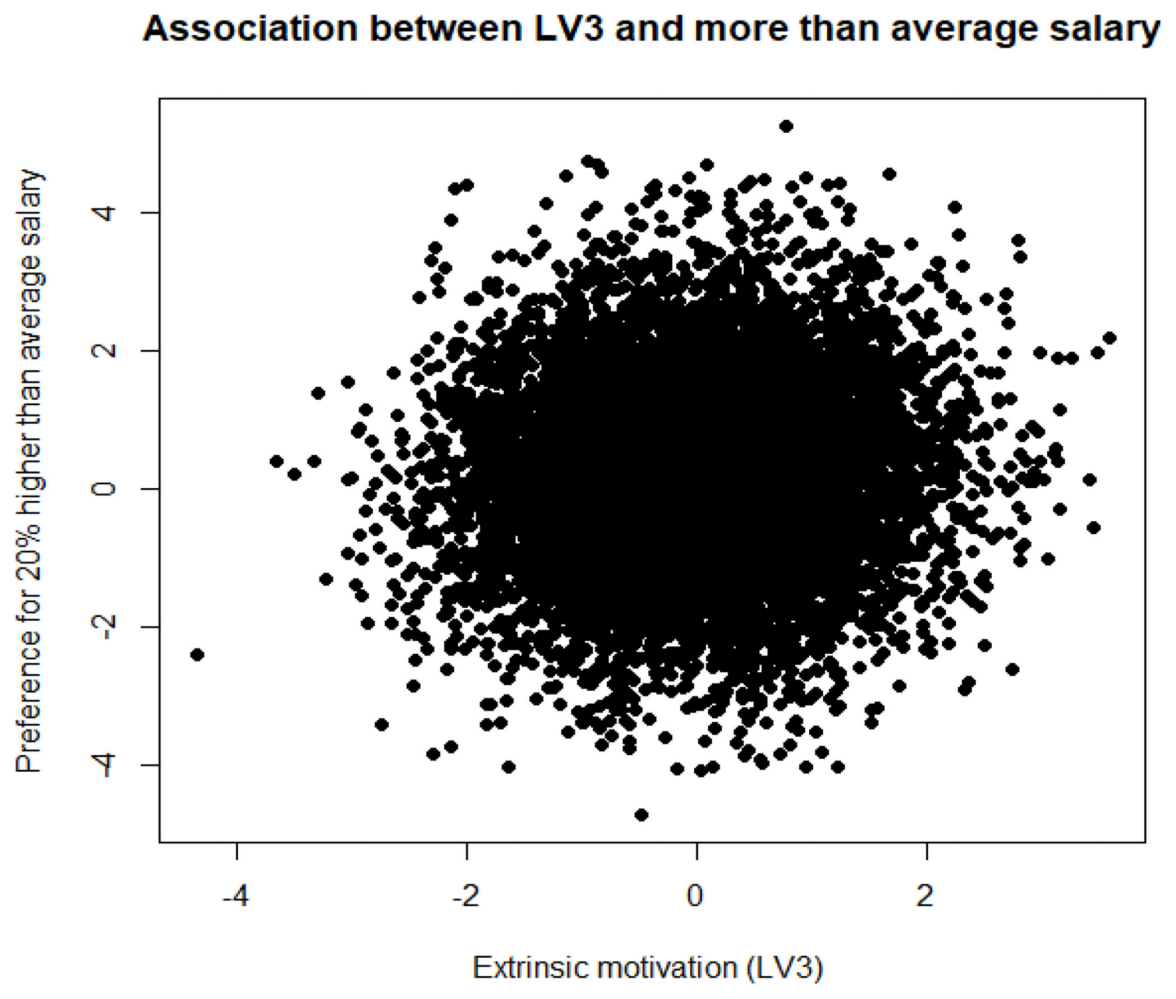
Association between extrinsic motivation and a higher than average salary

**Figure 6:**
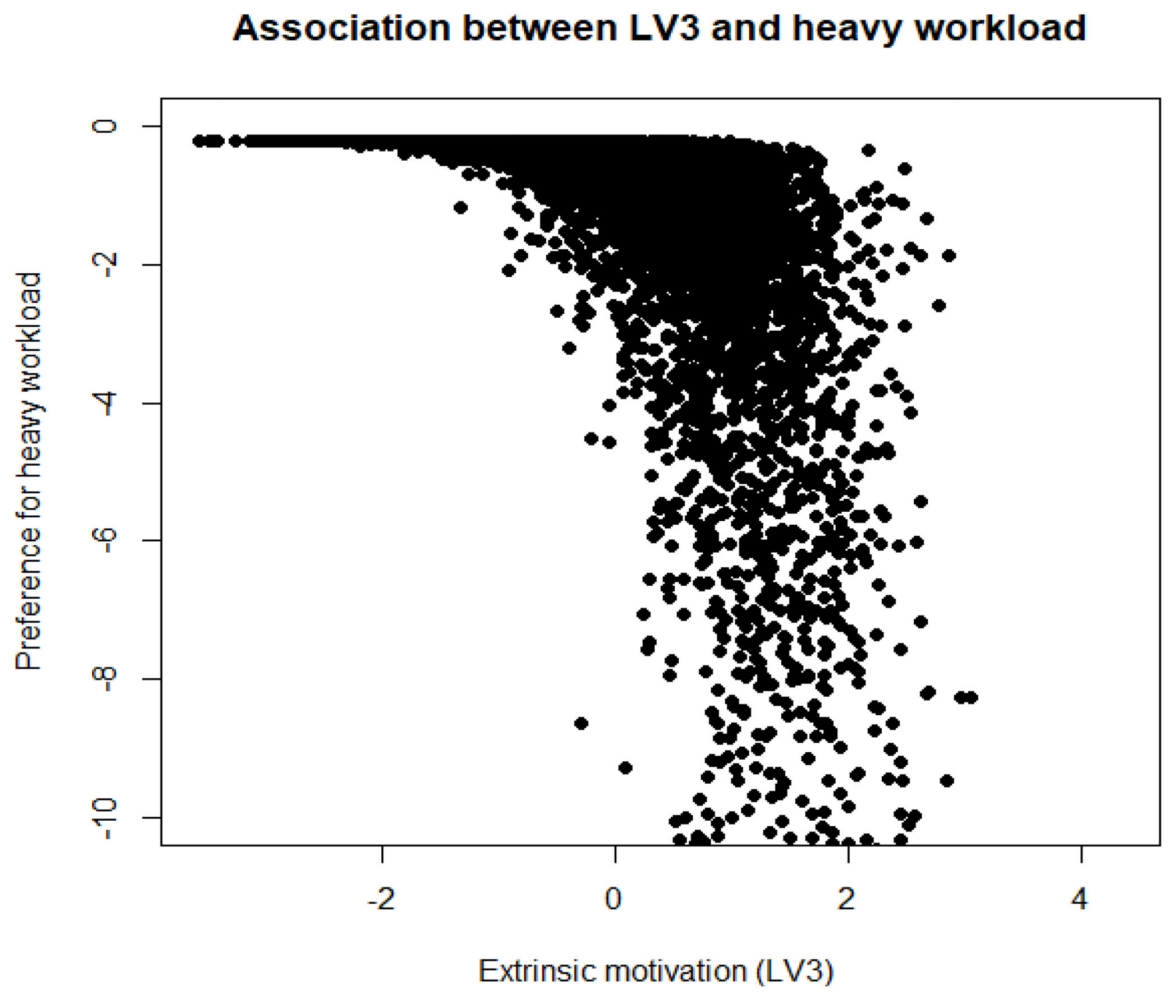
Association between extrinsic motivation and a heavy workload

Contrary to the above, extrinsically motivated HEWs show some preferences towards a higher than average salary (Figure 5), and strong dislike towards a heavy workload (Figure 6).

**Figure 7:**
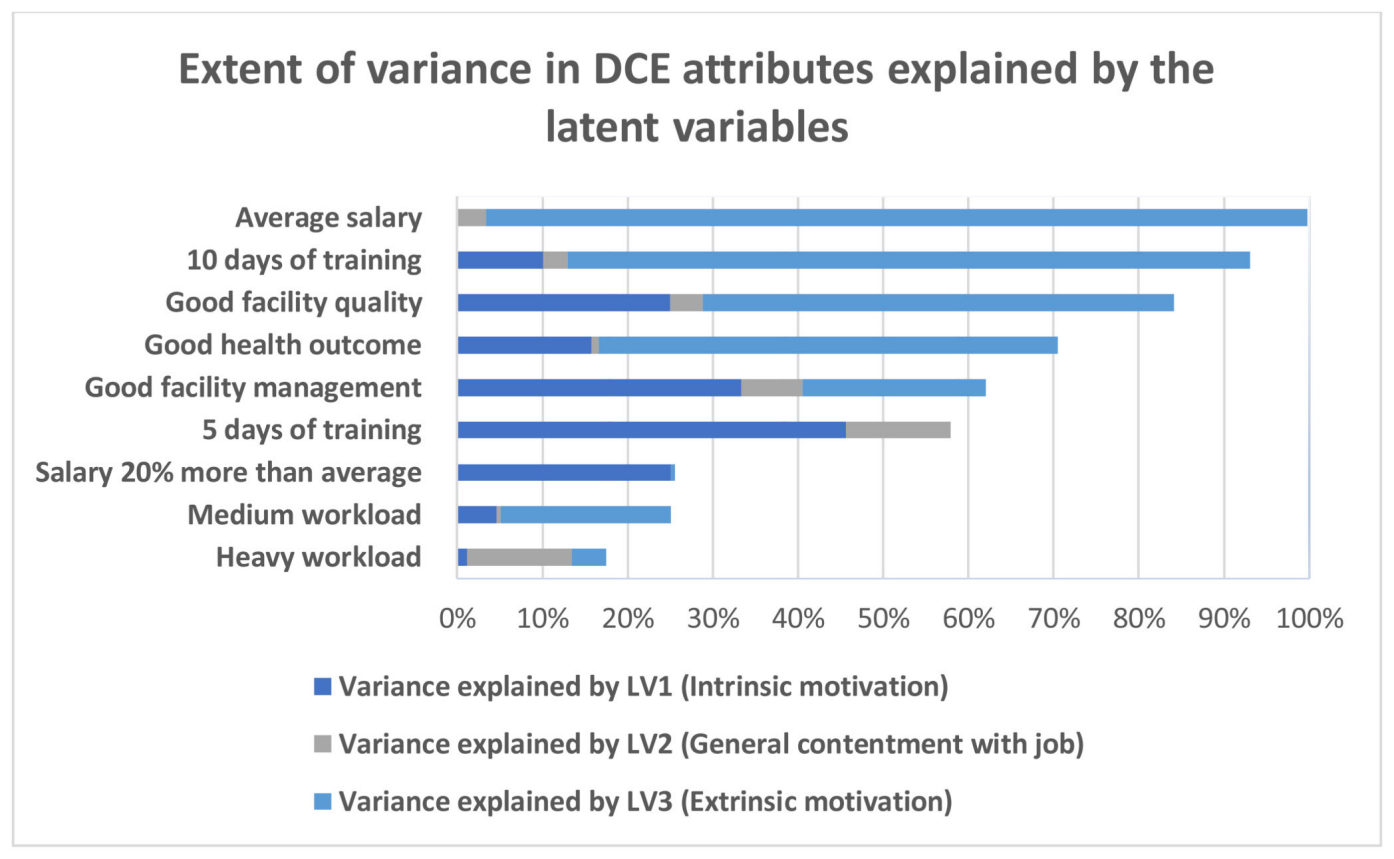
Extent of variation in preferences explained by the three latent variables

Lastly, Figure 7 shows the extent to which the variance in each attribute can be explained by all three latent variables. It can be seen that while a large proportion of variance for most of the attributes can be explained by latent variables 1 and 3 (intrinsic and extrinsic motivation, respectively), average salary and 10 days of training are especially worth noting as LV3 accounts for 96% and 80% variance, respectively. LV2 or general contentment with job contributes to a much lower proportion of preference variation contributed by motivation as a whole. It is important to note that the remaining heterogeneity in Figure 7 is random, and not linked to any of the latent variables in particular.

## 5 Discussion and Conclusions

Using data on CHWs in Ethiopia, we showed that health worker motivation is linked to their preferences for job attributes. We showed that for this analysis, when using motivation to explain heterogeneity in preferences, hybrid choice models outperform the models more traditionally used and allow us to overcome empirical concerns with endogeneity bias and measurement error (Buckell et al., 2021).

One of the key strengths of this study, in comparison to others on this topic, is that it explores the preferences and motivations of a group of lower-skilled frontline health workers who are central to the delivery of primary health care in Ethiopia and on whom there is little research. Our results show that HEWs that agreed to statements representing intrinsic motivation, also preferred jobs that offered lesser number of training days, opportunities to improve the health outcomes of people, and had supportive managers. They were neutral to a heavy workload, and disliked jobs with higher than average salaries. This is in line with theories on motivation which describe intrinsic motivation to be about a person’s desire to expend efforts based on their interest in and enjoyment of the activity itself (Gagné & Deci, 2005; Grant, 2008) rather than external rewards like salary. Further, substantiated by findings from our qualitative research with HEWs (Arora et al., 2020), we believe that a preference to spend lesser number of days on training is driven by their desire to not miss work for an extended period of time which can put them behind in the delivery of their tasks. We also found that HEWs with higher degrees were less likely to be intrinsically motivated. This was expected as better educated people in the labour market often tend to be driven by external rewards like higher remuneration for their work (Schweri & Hartog, 2015). Extrinsic motivation on the other hand refers to a person’s desire to make effort to obtain outcomes that are external to the activity itself and separable from it (Amabile, 1993; Brief & Aldag, 1977) so, people who tend to be extrinsically motivated are likely to be driven by things like their salary, praise from supervisors. Majority of our findings from the latent variable representing extrinsic motivation were not significant, however we did find that HEWs who were more likely to agree with statements representing extrinsic motivation also showed stronger disutility for a heavy workload and stronger preferences for a higher than average salary, which was in line with our expectations.

Our methods are subject to some limitations. First, we recognise that the quantitative tool on motivation was adapted from studies conducted in other countries which may have reduced its internal and external validity because Ethiopia has its distinct cultural, political, and health system landscape. To overcome this, we used expert opinion to ensure that the tool was tailored to the context, and then piloted it with health workers prior to rolling it out to make sure it is comprehendible. Second, while we have estimated a very detailed specification of the hybrid choice model with three latent variables, there are always opportunities for further developments. Our specification of the hybrid choice model focussed solely on motivation as the latent construct but there is clearly scope to also explore other latent components that could be present in the underlying structure of the model. Further, the reported association between the latent variables on choice behaviour should be interpreted with caution. Since motivation was not observed over time and the data was cross-sectional in nature, it is not clear if e.g. extrinsically motivated people preferred higher than average salaries or people who preferred higher salaries tended to be extrinsically motivated. Our intension was thus never to measure causality between motivation and job preferences, but to develop a model to estimate the extent of random variation that can be explained by motivation. Moreover, it was not clear which indicators from the tool on motivation should have been used to measure motivation in the hybrid choice model or whether to use the full set of available indicators. Using only some of the indicators risks overlooking key information, but on the other hand, using all indicators increases computational burden and can pose significant problems for modelling, because many indicators could be highly correlated which can leads to a proliferation of parameters and technical issues such as collinearity (Buckell et al., 2021). We, thus, included only statements with the highest factor loadings for each of the three latent variables, which we believed captured the key aspects of each dimension of motivation sufficiently. Third, due to correlation concerns, we had to reject Halton draws in favour of using 2000 MLHS draws (Bhat, 2003; Hess et al., 2006). We recognise that some recent literature does favour Sobol draws (Czajkowski & Budziński, 2019), however we thought the key point was to avoid Halton draws in this instance. Finally, due to our decision to include salary as a qualitative attribute, we were unable to include willingness-to-pay estimates in the study which could have provided useful information about how HEWs trade-off between individual attributes. It was not completely clear to us, why the *average salary* attribute in our models was not of the expected sign. Respondents may have read quickly and when they saw “20%” they assumed it was “20% higher than average”, not distinguishing between 20% higher and 20% lower. This would even be suggested by the results as there is no statistical difference between above-average and below-average (the omitted category) salaries. Without additional research and in the absence of qualitative evidence, however, it is not possible to know whether the validity of these parameter estimates is undermined.

Our approach to measure the labour market choices of community health workers using a hybrid choice approach provides promising results for choice modelers as well as managers and policy makers. These results are important from a behavioural and policy perspective as now we have more insight into the decision making processes, linking multidimensional motivation with job choices of health workers. These findings could be leveraged by managers and policy makers as psychometric tests, to assess the drivers of an individual in the labour workforce, are more commonly available and can be conducted ex ante to reveal their internal cognitive processes that make them expend effort towards a job. Future research linking other psychological processes like the knowledge and attitude of health workers, to the heterogeneity in their job preferences, is encouraged to further understand the factors that drive the decision making of frontline health workers.

## Supplementary Material

Supplementary file

## Figures and Tables

**Table 1:** Respondent characteristics

Variable description	Results
Respondent age (years)	Mean age (SD): 28 years (4.38)
Current monthly gross salary (ETB)	Mean (SD): ETB 3450 (24.72)
Months spent in the health system	Mean time spent in months (SD): 43 (.27)
Region of work	Tigray: 11.01%, Oromia: 24.95%, Amhara: 26.02%, SNNPR: 38.03%
Highest qualification attained	Level 1,2 or 3: 48.46%, Level4: 47.03%, degree or above: 4.5%
No of times the opt-out was chosen by respondents*	140 out of 1392 choice situations (10.06%)

ETB= Ethiopian Birr

* There were no serial non-respondents in the dataset who chose the opt-out for all 7 choice tasks

**Table 2:** DCE attributes and their levels

Attribute	Attribute levels
Salary	20% below average Average earnings 20% above average
Training	No training available 5 days per year dedicated training time (improving work-related and transferable skills) 10 days per year dedicated training time (improving work-related and transferable skills)
Workload	Light: more than enough time to complete duties Medium: enough time to complete duties Heavy: barely enough time to complete duties
Management style	Management is supportive, and makes work easier Management is not supportive, and makes work more difficult
Health facility quality	Your workplace is good: it has reliable electricity and other services, supplies are always available Your workplace is basic: it has unreliable electricity, whilst supplies you need are not always available
Opportunities to improve health outcomes	Your work will have a large impact on improving health in the local community Your work will have a small impact on improving health in the local community

**Table 3:** motivation statements included

Motivation statements	α	ζ	τ	Direction of association b/w for *I_k_n__* and *α_n,k_*
I am respected in my community for the work I do (positively framed)	1	1	1	opposite
My work is important because I help people (positively framed)	1	2	2	opposite
I can solve most problems I have at work if I work hard (positively framed)	1	3	3	opposite
I am proud of the work I do (positively framed)	2	4	4	opposite
In general I am satisfied with my role (positively framed)	2	5	5	opposite
At the moment I don’t feel like working as hard as I can (negatively framed)	3	6	6	same
I am strongly motivated by the recognition I get from other people (positively framed)	3	7	7	opposite

**Table 4:** Goodness of fit, mnl and mmnl models

Model	MNL	MMNL
Log likelihood	-1289.822	-1150.178
AIC	2601.64	2344.36
BIC	2659.32	2459.6
Number of parameters	11	22

**Table 5:** Estimation results of the hcm

Choice component log-likelihood	-1113.802		
Number of parameters	81		
Category	Parameter	Estimate	Rob.t ratio
Attribute mean (μ)	ASC 2	0.12955	1.43
	ASC 3	-4.75157**	-5.07
	Average salary	-0.41659**	-2.53
	20% more than average salary	0.26213	1.06
	5 days training	0.14988	0.46
	10 days training	-0.94624**	-3.13
	Medium workload	-0.39988	-1.23
	Heavy workload	-1.61824**	-2.81
	Good facility quality	0.44614**	2.12
	Good management	1.03045**	4.30
	Good outcome	-0.22582	-0.82
Attribute standard deviation (σ)	ASC 2	0.2136	-0.74
	ASC 3	4.00681**	4.96
	Average salary	0.0209	-0.15
	20% more than average salary	1.14456**	-3.49
	5 days training	0.99024**	-3.01
	10 days training	0.25803	0.50
	Medium workload	1.7077**	4.02
	Heavy workload	0.1402	0.85
	Good facility quality	0.64544**	-2.28
	Good management	0.41344*	1.87
	Good outcome	0.74496*	-1.90
**Latent Variable 1 - intrinsic motivation**			
Measurement Equations			
Interactions between α1 and choice model attributes (θ1)	ASC	-1.5222**	-2.78
	Average salary	0	NA
	20% more than average salary	0.67199*	1.86
	5 days training	-1.02352**	-2.27
	10 days training	-0.27666	-0.98
	Medium workload	0.42258	1.22
	Heavy workload	0.95491**	3.62
	Good facility quality	-0.74191**	-3.29
	Good management	-0.3876*	-1.66
	Good outcome	-0.51671**	-2.23
Impact of latent variables on motivationquestions (ζ)	ζ_m5_	3.70489**	2.88
	ζ_m22_	1.83052**	4.85
	ζ_m24_	1.82158**	4.58
**Latent variable 2 –General contentment with job**			
Measurement Equations			
Interactions between LV2 and choice model attributes (θ2)	ASC	0.37246	1.07
	Average salary	0.08053	0.59
	20% more than average salary	0	NA
	5 days training	-0.53012**	-2.28
	10 days training	0.14651	0.59
	Medium workload	-0.14496	-0.46
	Heavy workload	0.66867**	2.15
	Good facility quality	-0.28935	-1.47
	Good management	-0.17999	-1.12
	Good outcome	-0.1224	-0.51
Impact of latent variables on motivation questions (ζ)	ζ_m2_	-1.49849**	-4.40
	ζ_ml_	-5.71517	-1.50
**Latent variable 3 - Extrinsic motivation**			
Measurement equations			
Interactions between LV3 and choice model attributes (θ3)
	ASC	1.61086**	3.02
	Average salary	-0.434	-1.54
	20% more than average salary	0.09582	0.28
	5 days training	0	NA
	10 days training	-0.77831*	-1.87
	Medium workload	-0.88214**	-2.21
	Heavy workload	-1.76243**	-3.72
	Good facility quality	1.10404**	4.14
	Good management	0.31079	1.30
Good outcome	0.95504**	2.78
Impact of latent variables on motivation questions (ζ)	ζ_m6	0.67385**	2.73
	ζ_m3	0.65095**	3.74
τ_m5_1__	-	-1.77883	-2.89
τ_m5_2__	-	7.70711	3.27
τ_m5_3__	-	10.46417	2.98
τ_m22_1__	-	-1.65646	-5.55
τ_m22_2__	-	4.43378	6.42
τ_m22_3__	-	4.57458	6.69
τ_m22_4__	-	6.86627	5.80
τ_m24_1__	-	-1.50778	-5.17
τ_m24_2__	-	6.85885	5.68
τ_m1_1__	-	-1.87618	-6.39
τ_m1_2__	-	1.89631	5.97
τ_ml_3__	-	1.9793	6.17
τ_ml_4__	-	4.3585	6.99
τ_m2_1__	-	-4.54287	-1.64
τ_m2_2__	-	7.41804	1.70
τ_m2_3__	-	8.01774	1.68
τ_m2_4__	-	16.377	1.67
τ_m6_1__	-	-4.11719	-7.81
τ_m6_2__	-	0.12542	0.79
τ_m6_3__	-	0.23654	1.51
τ_m6_4__	-	3.8526	8.24
τ_m3_1__	-	-1.98732	-8.40
τ_m3_2__	-	1.11754	6.59
τ_m3_3__	-	1.29559	7.27
τ_m4_4__	-	5.48945	5.40

** significant at 5% level,

* significant at 10% level.

*Note:* The effects of certain latent variables on attributes were set to zero because they were very small and insignificant. These include the effect of LV1 on average salary, the effect of LV2 on 20% more than average salary, and the effect of LV3 on 5 days of training. As visible in this table, one tau per indicator variable for motivation was normalized

**Table 6:** factor analysis of motivation measure

statements	Factor 1 loadings	Factor 2 loadings	Factor 3 loadings
I am respected in my community for the work I do	0.72	-0.08	-0.12
My work is important because I help people	0.70	-0.13	-0.02
I can solve most problems I have at work if I work hard	0.69	-0.12	0.13
I am keenly aware of the career goals I have set for myself	0.67	0.04	-0.10
If I do well at work, I will achieve my goals	0.65	-0.01	0.04
It is important that I do a good job so that the health system works well	0.62	-0.07	0.20
Training sessions that I attend are worthwhile and add benefit to my career path	0.54	-0.11	0.23
I gain knowledge from being in this role	0.51	0.08	0.19
To be motivating, hard work must be rewarded with more status and money	0.51	-0.23	0.17
I can complete all of the work I am expected to do	0.50	0.12	-0.38
I feel committed to my role	0.48	0.10	-0.24
I feel like performing the duties required of me	0.48	0.20	-0.27
I am willing to do more than is asked of me in my role	0.39	0.23	0.16
I am proud to be working in my role	0.03	0.81	-0.06
In general I am satisfied with my role	0.02	0.67	0.00
I am proud of the work I do	0.24	0.64	0.12
The system of choosing who attends training sessions is fair	-0.06	0.44	0.03
My supervisors and managers are supportive of me	0.08	0.42	-0.03
My job makes me feel good about myself.	0.37	0.40	0.20
My work place provides everything I need to do my job properly	-0.13	0.39	-0.03
I am strongly motivated by the income I can earn at work	0.12	0.38	0.04
My salary accurately reflects my skills and workload	-0.15	0.36	0.22
At the moment I don’t feel like working as hard as I can	-0.01	-0.18	0.36
I am strongly motivated by the recognition I get from other people	0.22	0.18	0.42

Figure notes: the statements in grey loaded to factor 1, statements in blue loaded to factor 2, and the statements in green loaded to factor3
