# Supplementary material for: Linking health worker motivation with their stated job preferences: A hybrid choice analysis in Ethiopia
Source: Soc Sci Med. Author manuscript; Available in PMC 2023 Aug 4. (PMC7614882; doi:10.1016/j.socscimed.2022.115151)
Supplement: Supplementary file [file EMS181741-supplement-Supplementary_file_.docx]

# **Supplementary file**

Table 1 gives the results of the factor analysis, including all 30 statements on motivation.

Table 1: Results of the factor analysis

| **Statements** | **Factor 1 loadings** | **Factor 2 loadings** | **Factor 3 loadings** |
| --- | --- | --- | --- |
| I am respected in my community for the work I do | 0.72 | -0.08 | -0.12 |
| My work is important because I help people | 0.70 | -0.13 | -0.02 |
| I can solve most problems I have at work if I work hard | 0.69 | -0.12 | 0.13 |
| I am keenly aware of the career goals I have set for myself | 0.67 | 0.04 | -0.10 |
| If I do well at work, I will achieve my goals | 0.65 | -0.01 | 0.04 |
| It is important that I do a good job so that the health system works well | 0.62 | -0.07 | 0.20 |
| Training sessions that I attend are worthwhile and add benefit to my career path | 0.54 | -0.11 | 0.23 |
| I gain knowledge from being in this role | 0.51 | 0.08 | 0.19 |
| To be motivating, hard work must be rewarded with more status and money | 0.51 | -0.23 | 0.17 |
| I can complete all of the work I am expected to do | 0.50 | 0.12 | -0.38 |
| I feel committed to my role | 0.48 | 0.10 | -0.24 |
| I feel like performing the duties required of me | 0.48 | 0.20 | -0.27 |
| I am willing to do more than is asked of me in my role | 0.39 | 0.23 | 0.16 |
| I am proud to be working in my role | 0.03 | 0.81 | -0.06 |
| In general I am satisfied with my role | 0.02 | 0.67 | 0.00 |
| I am proud of the work I do | 0.24 | 0.64 | 0.12 |
| The system of choosing who attends training sessions is fair | -0.06 | 0.44 | 0.03 |
| My supervisors and managers are supportive of me | 0.08 | 0.42 | -0.03 |
| My job makes me feel good about myself. | 0.37 | 0.40 | 0.20 |
| My work place provides everything I need to do my job properly | -0.13 | 0.39 | -0.03 |
| I am strongly motivated by the income I can earn at work | 0.12 | 0.38 | 0.04 |
| My salary accurately reflects my skills and workload | -0.15 | 0.36 | 0.22 |
| At the moment I don’t feel like working as hard as I can | -0.01 | -0.18 | 0.36 |
| I am strongly motivated by the recognition I get from other people | 0.22 | 0.18 | 0.42 |

Figure notes: the statements in grey loaded to factor 1, statements in blue loaded to factor 2, and the statements in green loaded to factor3

Table 2 presents estimation results of the main effect MNL model.

Table 2: Estimation results of MNL

| **Parameter** | **Estimate** | **Rob.s.e.** | **Rob.t.ratio** |
| --- | --- | --- | --- |
| ASC 2 | 0.06721 | 0.04716 | 1.4251 |
| ASC 3 | -1.32642 | 0.22736 | -5.8341 |
| Average salary | -0.14423 | 0.08335 | -1.7305 |
| 20% more than average salary | 0.22427 | 0.11645 | 1.9258 |
| 5 days training | 0.1348 | 0.14404 | 0.9358 |
| 10 days training | -0.39102 | 0.14498 | -2.6971 |
| Medium workload | -0.18338 | 0.1442 | -1.2717 |
| Heavy workload | -0.49583 | 0.10057 | -4.9303 |
| Good facility quality | 0.23313 | 0.08588 | 2.7147 |
| Good management | 0.56187 | 0.10043 | 5.5948 |
| Good outcome | -0.10102 | 0.13062 | -0.7734 |

Table 3 gives the estimation results of the main effects MMNL model.

Table 3: Estimation results of the MMNL

| **Category** | **Parameter** | **Estimate** | **Rob.s.e.** | **Rob.t.ratio** |
| --- | --- | --- | --- | --- |
| Attribute mean (μ) | ASC 2 | 0.102081 | 0.0703 | 1.45216 |
|  | ASC 3 | -4.279222 | 0.67666 | -6.32403 |
|  | Average salary | -0.412419 | 0.14759 | -2.79434 |
|  | 20% more than average salary | 0.372877 | 0.21013 | 1.77454 |
|  | 5 days training | 0.110371 | 0.28292 | 0.39011 |
|  | 10 days training | -1.087717 | 0.27811 | -3.9111 |
|  | Medium workload | -0.235479 | 0.28526 | -0.8255 |
|  | Heavy workload | -1.314493 | 0.37711 | -3.48574 |
|  | Good facility quality | 0.267694 | 0.12764 | 2.09729 |
|  | Good management | 0.962115 | 0.17775 | 5.41281 |
|  | Good outcome | -0.336504 | 0.24027 | -1.40054 |
| Attribute standard deviation (σ) | ASC 2 | -0.002088 | 0.03239 | -0.06446 |
|  | ASC 3 | 3.365752 | 0.3891 | 8.65011 |
|  | Average salary | -0.009091 | 0.03241 | -0.28051 |
|  | 20% more than average salary | -0.782301 | 0.22273 | -3.5123 |
|  | 5 days training | -0.350515 | 0.41888 | -0.83679 |
|  | 10 days training | -0.636157 | 0.43043 | -1.47796 |
|  | Medium workload | 2.091217 | 0.32716 | 6.39211 |
|  | Heavy workload | 1.589823 | 0.40046 | 3.97001 |
|  | Good facility quality | -0.849086 | 0.1693 | -5.01541 |
|  | Good management | 0.020835 | 0.05456 | 0.38187 |
|  | Good outcome | 0.441759 | 0.42974 | 1.02796 |
